# Prevalence of fat by cardiac magnetic resonance imaging stratified by age in 940 patients referred for evaluation of arrhythmogenic (rv) dysplasia

**DOI:** 10.1186/1532-429X-13-S1-P306

**Published:** 2011-02-02

**Authors:** Hector Medina-Zuluga, Daniel Verdini, Manavjot Sidhu, Peerawut Deeprasertkul, Yongkasem Vorasettakarnkij, Waleed Ahmed, Thomas J Brady, Thomas Neilan, Stephan B Danik, Suhny Abbara, David E Sosnovik, Gotfred Holmvang, Brian Ghoshhajra

**Affiliations:** 1 Mass General Hospital and Harvard School of Medicine, Boston, MA, USA

## Objective

To determine the prevalence and severity of RV fatty infiltration, stratified by age, in patients referred to cardiac MRI (cMRI) for suspected Arrhythmogenic Right Ventricular Dysplasia (ARVD).

## Background

cMRI is frequently used to assess right ventricular (RV) morphology and function in patients with suspected ARVD and is the gold standard for fatty metaplasia amongst all non-invasive imaging tests. However, the incidence and severity of RV fatty infiltration by cMRI is not well defined. Here, stratified by age, we investigate the prevalence and severity of RV fat infiltration in a large population undergoing dedicated imaging for fat detection.

## Methods

All consecutive patients referred for cMRI with suspected ARVD between 2003-2010 in our institution were included. RV fat was determined by cine-balanced SSFP, gradient-echo, and spin-echo sequences with and without fat suppression. RV fat severity was graded as mild, moderate or severe depending on the extension of its distribution. Age was stratified in tertiles (T3 oldest, T2 middle and T1 youngest) and fatty infiltration severity in each of these age sub-groups was analyzed.

## Results

**Figure 1:**
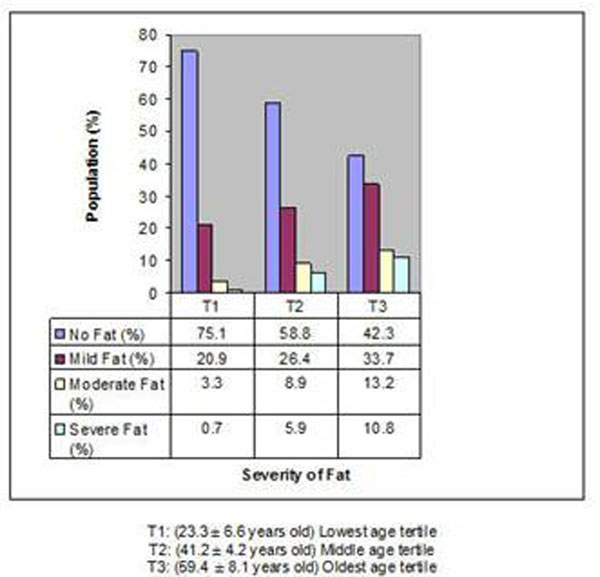
Prevalence of Fat by Cardiac MRI in Patients Referred for Evaluation of ARVD

A total of 940 patients were included in our study. Patients with any fat (n=392, 41.7%) in the RV tended to be older compared to patients with no fat (mean age 47.5 ± 14.3 vs. 37.8 ± 16.3, p < 0.0001, respectively). Patients in T3 tended to have a higher proportion of severe fat in the RV compared to patients in T2 (10.8% vs. 5.9%, p =0.03) and T1 (10.8% vs. 0.7%, p < 0.0001). Also, patients in T3 tended to have higher proportion of moderate fat in the RV compared to patients in T2 (13.2% vs. 8.9%, p =0.09) and T1 (13.2% vs. 3.3%, p<0.001). Finally, T3 patients tended to have the lowest proportion of RV without fat compared to patients in T2 and T1 (42.3% vs. 58.8 % vs. 75.1%, respectively; p < 0.0001). Full fat and age distribution by tertiles are depicted in Fig 1.

## Conclusions

The prevalence of RV fat during cMRI in patients referred for suspected ARVD is high and older patients tend to the highest prevalence of fat and higher proportions of severe and moderate fat distribution. Our findings suggest that the presence RV fat above the age of 60 may represent a common finding related with aging whereas the same finding in the first two decades of life is uncommon and may have a higher diagnostic yield for ARVD.

